# Effect of Water Stress on Physiological and Morphological Leaf Traits: A Comparison among the Three Widely-Spread Invasive Alien Species *Ailanthus altissima*, *Phytolacca americana,* and *Robinia pseudoacacia*

**DOI:** 10.3390/plants11070899

**Published:** 2022-03-28

**Authors:** Maria Pepe, Maria Fiore Crescente, Laura Varone

**Affiliations:** Department of Environmental Biology, Sapienza University of Rome, P. le A. Moro 5, 00185 Rome, Italy; maria.pepe@uniroma1.it (M.P.); mariafiore.crescente@uniroma1.it (M.F.C.)

**Keywords:** invasive alien species, water stress, leaf water status, gas exchange, LMA, stomatal limitations

## Abstract

Invasive alien species (IAS) are a problem, especially in drought-prone environments such as the Mediterranean Basin where the exacerbation of the already severe conditions could constrain the native species acclimatation degree, creating new opportunities for IAS. Climate change may drive IAS expansions, even if different IAS can vary in their acclimatation response. Thus, it is important to obtain a broader insight of how the different IAS face abiotic stress. This research aimed to compare the effect of the imposed water stress on physiological and morphological leaf traits of *Ailanthus altissima* (AA), *Robinia pseudoacacia* (RP), and *Phytolacca americana* (PA), which are widely spread IAS in the Mediterranean Basin. Our results showed a species-dependent effect of the water stress at a physiological and morphological level, as well as an interaction between species and stress duration. Despite a common strategy characterized by low stomatal control of the photosynthesis, AA, PA, and RP differ in their sensitivity to water stress. In particular, even if AA was characterized by a more water-spending strategy, it was more resistant to water stress than PA and RP. In this view, the key factor was its plasticity to increase leaf mass per area (LMA) in response to water stress.

## 1. Introduction

The naturalization process of plant species in a territory outside their native distribution range results from the combination of different taxonomic, biogeographic, climatic, edaphic, and economic factors [1,2]. Conservative estimates suggest that at least 3.9% of global vascular flora have been successfully naturalized in newly introduced regions [3,4]. Most of these naturalized alien species do not produce harmful effects on native species [1,5,6]. However, about 1% of the introduced alien species becomes invasive [3,4], leading to a threat to the local plant diversity, with severe consequences on ecosystem functioning [7,8,9].

Invasive alien species (IAS) represent a problem, especially in drought-prone environments such as the Mediterranean Basin, which is subject to severe ecological conditions [10,11,12,13]. Currently, climate shift models forecast a worsening of the stressful conditions in the Mediterranean Basin, where prolonged drought phenomena are already occurring [14,15,16]. In this context, global climate change is expected to reduce the degree of adaptation of numerous native species to the environment, creating new opportunities for IAS [17,18]. There is a large consensus in considering native plants as generally less tolerant to stresses than IAS, thus having a lower plasticity in acclimating to environmental changes [19,20,21,22,23]. Besides climatic factors, the vulnerability of the Mediterranean Basin region to IAS is also due to a high urbanization level and natural resources exploitation, which are recognized as drivers for IAS spreading [24]. As further proof of this, under current and future climatic conditions, the Mediterranean Basin zone, including Europe and Anatolia, which is also the most urbanized, is particularly considered at a high risk of IAS establishment [10,12,25,26].

The capacity of IAS to modulate functional traits in response to environmental changes is particularly useful in environments such as Mediterranean ones, characterized by seasonal resource fluctuations, as they allow IAS either to successfully exploit the resource surplus under high availability conditions or to use resources more efficiently in limiting conditions [27,28,29,30]. At a physiological level, studies on IAS capacity for acquiring resources showed that their competitive advantage is usually associated with a higher CO_2_ assimilation rate [31,32,33] and is supported by an increased photosynthetic pigment and carbohydrate content [34,35]. Indeed, these factors create conditions for IAS to rapidly capture resources and space and thus outcompete native species [36]. Photosynthesis may be considered a highly responsive variable able to rapidly point up the functional limitations imposed by the environment [37]. Moreover, due to its interaction with a range of physiological and morphological traits underlying plant growth, photosynthesis may be used as a proxy for the ability of species to adapt to the local climate [38]. Under a Mediterranean type climate, the photosynthetic CO_2_ uptake can be seriously constrained by prolonged drought periods.

Mechanisms underlying photosynthesis impairment caused by water stress consist of diffusive limitations, which act at stoma and mesophyll levels, and biochemical limitations, which occur at a chloroplast level [39]. In particular, metabolic limitations lead definitively to a photosynthetic down-regulation because of constraints to Rubisco activity and RuBP regeneration capacity [11,18,40,41,42,43]. The relative importance of the diffusive and metabolic limitations is related to the stress intensity and duration. Usually, in response to low or moderate stress, stomatal limitations prevail on metabolic ones, which become more important under severe water stress [44,45,46]. In particular, improved water use efficiency (WUE) under drought is achieved by a reduced stomatal aperture for a given carbon assimilation rate, which has been shown to occur as a result of both adaptation and acclimation processes [47,48]. A greater competitive ability of IAS could include a greater use of limiting resources, such as water, thereby inhibiting the establishment, survival, and reproduction of native species [49]. In general, a study showed that IAS had a greater WUE, and adaptations at root level allow a more efficient water uptake [50]. However, these adaptations may vary according to the species and the environment in which they are established [50].

Plants develop a wide range of mechanisms in response to drought [51]. In particular, a leaf is the organ that promptly responds to environmental conditions [52]; thus, its structure may reflect the effects of water stress more clearly in respect to other organs such as the stem and roots [53].

At a morphological level, protection against drought is conferred by leaf mass per area (LMA), which is strictly related to mechanistic functional traits such as photosynthesis and leaf transpiration [54].

LMA shows a high responsiveness in response to light conditions, soil fertility, and especially to drought [55] by modification of its components such as leaf thickness and leaf tissue density [43,56].

The tendency for LMA to increase with decreasing water availability is well known [50,57]. Indeed, high LMA is a recurrent leaf trait of Mediterranean species [55,58], with a special protection function for plants facing long periods of drought stress [11].

Thus, changes in LMA allow to expand the knowledge on physiological function involved in the acclimation process to environmental stress conditions.

Over the years, ecological studies have been mainly focused on the acclimatation capacity of IAS to environmental factor changes compared to native species [59,60,61,62]. These studies contributed to elucidate the mechanisms underlying the invasion process [63,64]. Although it is considered that climate change is a driver for future IAS expansions [64,65,66,67,68], the degree of acclimation to new and more severe conditions may considerably vary between different IAS. Accordingly, it would be strategically important to expand knowledge on the interspecific variability in responding to abiotic stress, such as water deficiency, to obtain a broader insight on the IAS capacity to further expand their areal under new climatic conditions.

Acclimation capacities based on water conservation strategies are of paramount importance to preserve the functionality of the photosynthetic apparatus allowing species to maintain efficient photosynthetic rates [69,70,71]. Thus, analyzing the effect of drought on diffusive and metabolic traits related to carbon gain can be a useful approach to test the IAS plasticity to stress factors [72,73] and therefore their capability to persist and/or further expand their distribution in the Mediterranean Basin.

Among woody IAS invading temperate and Mediterranean ecosystems, *Ailanthus altissima* (Mill.) Swingle is one of the most successful competitors with native vegetation [69]. *Ailanthus altissima* is a deciduous tree native to China, growing naturally in subtropical/warm temperate climates. It was introduced in Europe and North America in the 18th century, and now it is considered one of the worst invasive plant species [24,74]. Generally, it prefers environments altered by human activities, such as cities, transportation corridors, and agricultural fields. *Ailanthus altissima*, a shade-intolerant species, is highly tolerant to abiotic stress including drought [69,75], showing adaptations such as a high reduction of the water loss by leaves and a simultaneous reduction in root hydraulic conductance [69].

*Ailanthus altissima* is often associated with *Robinia pseudoacacia* L., another important IAS [75]. *Robinia pseudoacacia* is a fast-growing tree species native to two separate areas in the south-eastern USA, with the distribution center in the Appalachian Mountains [76,77]. In its native range, *Robinia pseudoacacia* grows best at sites characterized by a humid climate [76,78]. However, *Robinia pseudoacacia* is an extremely ecologically plastic species. It also adapts well to precipitation/groundwater conditions through the anatomy of wood, and its growth is limited by shade [79]. However, climate change may cause significant losses in the availability of niches for this species in southern Europe [80].

Among the herbaceous IAS, one of the most widespread in Mediterranean areas is *Phytolacca americana* L., which is usually associated with *Robinia pseudoacacia* plantations [81]. *Phytolacca americana* is a perennial plant native to the eastern part of North America. In its native range, it primarily grows as a pioneer plant of disturbed and open surfaces of damp soiled forests, on the fringe of forests, and on riverbanks [81]. Generally, *Phytolacca americana* prefers humid habitats and the half-shade [81].

Accordingly, the objective of this research was to compare physiological and morphological responses of *Ailanthus altissima*, *Robinia pseudoacacia*, and *Phytolacca americana*, three species cooccurring in the city of Rome, to experimentally imposed water stress. Moreover, considering the native climate and occupied habitats of the considered species, we wanted to test the hypothesis that *Ailanthus altissima* had a greater resistance to aridity and could therefore show a better response to imposed water stress.

## 2. Results

### 2.1. Leaf Water Status

**Figure 1:**
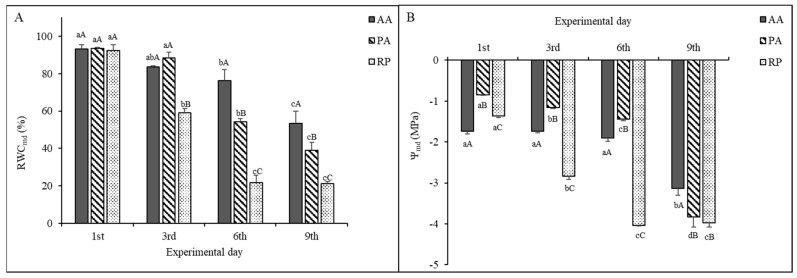
(**A**) Midday relative content (RWC_md_) and (**B**) midday leaf water potential (Ψ_md_) of *Ailanthus altissima* (AA), *Phytolacca americana* (PA), and *Robinia pseudoacacia* (RP) during the study period. Measurements carried out on the first experimental day (i.e., 1st) corresponded to control values. Mean ± standard deviation is shown (bars). Lowercase letters indicate differences within species, capital letters indicate differences among species. The means with the same letters are not significantly different (two-way ANOVA, *p* ≤ 0.005).

Two-way ANOVA revealed that RWC_md_ and ψ_md_ responses to water stress were significantly affected by species, sampling day, and the interaction of the two factors (Table 1). On the first day of the experiment, when all plants were well watered, differences in RWC_md_ between the considered species were not significant (Figure 1A), with values ranging from 94 ± 1% (PA) to 92 ± 3 (RP). On the contrary, significant differences were observed in ψ_md_ (Figure 1B), with AA showing the most negative values (−1.74 ± 0.06 MPa) followed by RP (−1.37 ± 0.03 MPa) and PA (−0.85 ± 0.2 MPa). Both RWC_md_ and ψ_md_ differently decreased among AA, PA, and RP throughout the experiment. RP showed a significantly large RWC_md_ and ψ_md_ decrease already after three days of water stress, reaching dramatic low values on the sixth sampling day (RWC_md_ = 22 ± 4%, ψ_md_ = −4.04 MPa ± 0.01 MPa).

In AA, not significant differences in RWC_md_ and ψ_md_ were found between the first and third sampling day, whereas six days of water withholding lead to a slight but significant decrease in RWC_md_ but not in ψ_md_. Leaf water parameters substantially decreased on the ninth sampling day (RWC_md_ = 53 ± 6%, ψ_md_ =−3.14 ± 0.17 MPa). PA showed a similar trend to AA with regard to RWC_md_, even if the extent of the reduction was higher in PA than AA. As for ψ_md_, PA showed a temporal dynamic similar to RP with a significantly lower value than the first day, starting from the third sampling day. However, unlike RP, PA reached very low values (3.83 ± 0.25 MPa) after nine days of water stress.

### 2.2. Gas Exchange Measurements

Gas exchange parameters, similar to leaf water status parameters, significantly varied between species and sampling days, with a significant species × sampling day interaction (Table 1).

**Figure 2:**
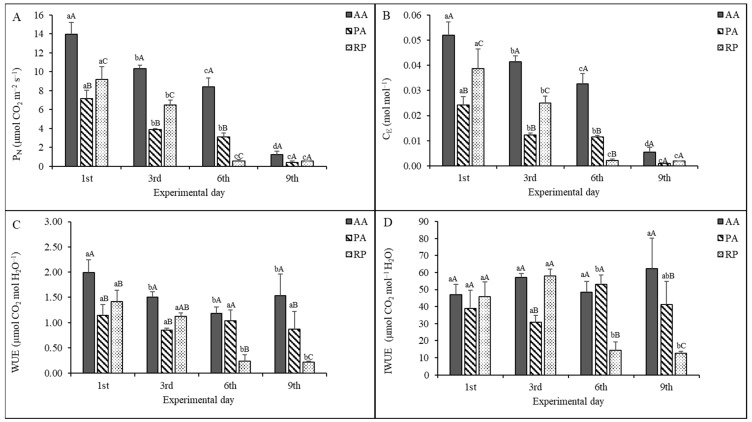
(**A**) Net photosynthetic rate (P_N_), (**B**) apparent carboxylation efficiency (C_E_), (**C**) water use efficiency (WUE), and (**D**) intrinsic water use efficiency (IWUE) of *Ailanthus altissima* (AA), *Phytolacca americana* (PA), and *Robinia pseudoacacia* (RP) during the study period. Measurements carried out on the first experimental day (i.e., first) correspond to control values. Mean ± standard deviation is shown (bars). Lowercase letters indicate differences within species, and capital letters indicate differences among species. The means with the same letters are not significantly different (two-way ANOVA, *p* ≤ 0.005).

In the well-watered condition, P_N_ (Figure 2A) was the highest in AA (14.0 ± 1.3 µmol CO_2_ m^−2^s^−1^), followed by RP (9.2 ± 1.3 µmol CO_2_ m^−2^s^−1^) and PA (7.1 ± 0.9 µmol CO_2_ m^−2^s^−1^). Across the sampling days, AA showed a slower P_N_ decrease than PA and RP. After six days of water stress, P_N_ was 39%, 54%, and 95% lower than the first day in AA, PA, and RP, respectively. Indeed, in AA and PA, a further P_N_ decrease was observed on the ninth day of water stress, with values 91% and 94% lower than the first day.

C_E_ substantially followed the P_N_ trend (Figure 2B), as it decreased at the comparable extent across the species and sampling day. Indeed, on the sixth day, C_E_ was 37%, 52%, and 95% lower than the first day in AA, PA, and RP, respectively.

Unlike P_N_ and C_E_, WUE and IWUE showed a lower responsiveness to water stress in all species, especially in PA, that did not significantly vary WUE across the sampling day (Figure 2C) and in AA, which maintained a stable IWUE (Figure 2D).

In AA, WUE significantly decreased on the third day of water stress (WUE = 1.50 ± 0.12 µmol CO_2_ mol H_2_O^−1^) compared to the first sampling day (1.99 ± 0.25 µmol CO_2_ mol H_2_O^−1^). However, since the third day, WUE did not significantly change longer throughout the experiment. RP showed a significant WUE decrease only on the sixth day (83% lower than the first day). In PA, water withholding for six days led to significantly increased IWUE by 36% compared to the no stressed condition when IWUE was equal to 39 ± 10 µmol CO_2_ mol^−1^ H_2_O. The first three days of water stress did not produce an effect on the IWUE of RP, which on the sixth day unexpectedly decreased IWUE by 69% compared to the first day.

### 2.3. Leaf Morphology

Two-way ANOVA revealed that the effect of water stress on morphological traits was dependent on the species and sampling day, without any significant interaction effect between the two factors (Table 2).

AA showed the largest LA (136.58 ± 25.5 cm^2^) and LMA (6.11 ± 0.98 mg cm^−2^) under well-watered conditions. No significant differences were found in LA and LMA between PA and RP, which showed values about two-fold lower than AA. In AA, water stress induced a significant 22% decrease of LA, as well as a 23% LMA increase. No significant differences between unstressed and stressed conditions were found in PA and RP.

### 2.4. Pearson’s Correlation Analysis and Treatment Effect Size

**Figure 3:**
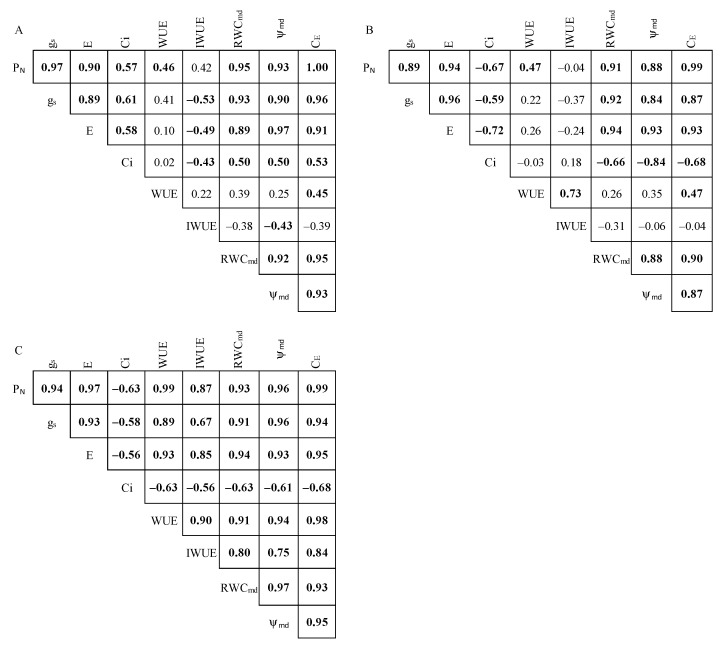
Correlation matrix of (**A**) *Ailanthus altissima* (AA), (**B**) *Phytolacca americana* (PA), and (**C**) *Robinia pseudoacacia* (RP). Net photosynthetic rate (P_N_), stomatal conductance (g_s_), transpiration rate (E), sub-stomatal CO_2_ concentration (C_i_), water use efficiency (WUE), intrinsic water use efficiency (IWUE), midday relative water content (RWC_md_), midday leaf water potential (ψ_md_), and apparent carboxylation efficiency (C_E_). Bold values indicate a significant correlation (*p* < 0.05).

**Figure A1:**
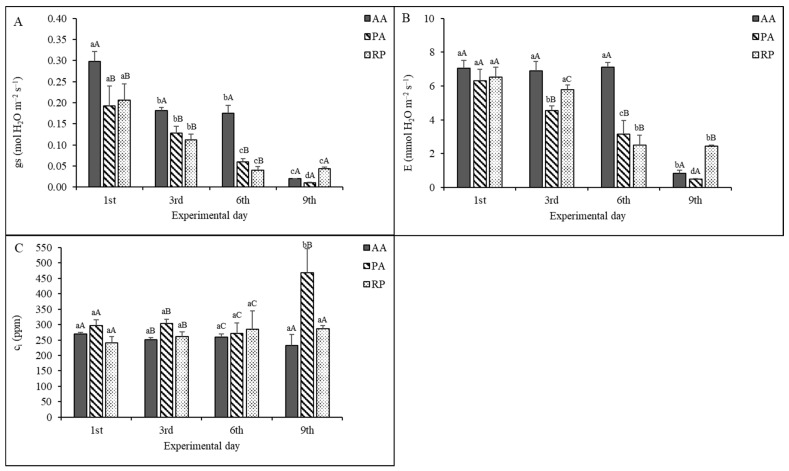
(**A**) Stomatal conductance (g_s_), (**B**) transpiration rate (E), and (**C**) sub-stomatal CO_2_ concentration (C_i_) of *Ailanthus altissima* (AA), *Phytolacca americana* (PA), and *Robinia pseudoacacia* (RP) during the study period. Measurements carried out on the first experimental day (i.e., first) correspond to the control values. Mean ± standard deviation is shown (bars). Lowercase letters indicate differences within species, and capital letters indicate differences among species. The means with the same letters are not significantly different (two-way ANOVA, *p* ≤ 0.005).

The Pearson’s linear correlation analysis was carried out by using all physiological traits, also including g_s_, E, and C_i_ (the values of these latter are shown in Appendix A, Figure A1). The Pearson’s analysis confirmed the existence of a strong and significant relationship among the considered physiological traits (Figure 3).

Nevertheless, some differences among the species were observed. In RP, the variation of WUE and IWUE significantly depended on the variation in P_N_, g_s_, and E. In AA, the imposed water stress caused significant WUE decrease, which resulted mainly from P_N_ decrease rather than from E reduction, as attested by the significant correlation between P_N_ and WUE. On the contrary, no correlation was found between E and WUE.

Moreover, the low stomatal control on P_N_ in AA was also highlighted by the lack of significant correlation between P_N_ and IWUE.

In PA, IWUE was not correlated with P_N_, g_s_, and E, while WUE was correlated with P_N_ but not with g_s_ and E. In all species, gas exchange traits were also strongly correlated to ψ_md_ and RWC_md_.

**Figure 4:**
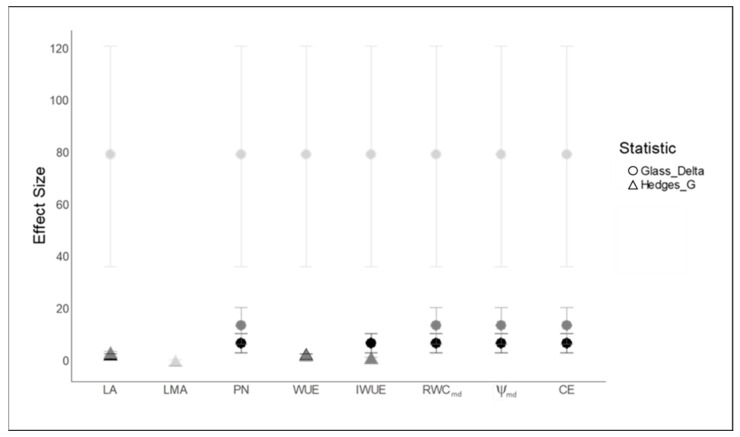
Effect size of the experimental treatments over trait expression. Leaf area (LA), leaf mass per area (LMA), net photosynthetic rate (P_N_), water use efficiency (WUE), intrinsic water use efficiency (IWUE), midday relative water content (RWC_md_), midday leaf water potential (ψ_md_), and apparent carboxylation efficiency (C_E_). Dots and error bars are Hedges’ g and Glass’s delta and 95% confidence intervals, averaged for the several species and water stress statuses. The effect size refers to the responses between the control (i.e., first) and the end of the experiment (i.e., ninth). *Ailanthuss altissima* (AA) (black symbols), *Phytolacca americana* (PA) (grey symbols), and *Robinia pseudoacacia* (RP) (light grey symbols).

Treatment effect sizes (i.e., Glass’s delta) concerning RWC_md_ and ψ_md_ were larger for RP (Figure 4). Moreover, RP showed a larger treatment effect size (i.e., Hedges’ g and Glass’s delta) also with regard to physiological traits. On the other hand, treatment effect size on morphological traits (i.e., Hedges’ g) differed between LA and LMA. In particular, RP was characterized by a larger Hedges’ g, relative to LA, whereas the effect size of LMA did not differ among species.

## 3. Discussion

Water stress alters plant functions by eliciting concurrent changes in physiological, morphological, and biochemical traits. The extent to which the functional traits differ between species depends on their capacity to tolerate or not tolerate water stress. The intensity and duration of water stress also contributed to discrimination between species. In addition, although the imposed water stress experiment did not last long, the intensity was such as to induce, in some species, significant variations in morphological traits.

During the first six days of water stress, AA kept a more favorable leaf water status, maintaining unchanged both ψ_md_ and RWC_md_, which dramatically decreased after nine days of water withholding. RWC and ψ_md_ are sensitive indicators of short-term leaf adjustments to face water stress. Among morphological adjustments, a larger LMA being usually associated with a high leaf thickness and/or density [43,82,83,84,85,86], enhanced the resistance to water stress [42,85,87]. Leaves with a larger LMA allowed to maintain a higher tissue water and therefore a high RWC [88]. Among the considered species, AA showed the largest LMA both under well-watered and stressed conditions. Moreover, AA also showed a great responsiveness in increasing LMA, as stressed seedlings at the maximum water stress, i.e., on the ninth day, were characterized by a significant 22% larger LMA than unstressed ones. Thus, the capacity of AA to keep a fairly stable RWC_md_ for several days could account for its larger LMA.

A better leaf water status during the first six days of water stress allowed AA to photosynthesize at rates ranging from 74% to 60% of the maximum rates coinciding with well-watered conditions. Starting from the sixth day, a strong P_N_ impairment occurred simultaneously with the maximum water stress, leading to lethal values. Indeed, when photosynthetic rates drop below 70% of the maximum, the P_N_ recovery of unstressed values may be compromised [86,89]. Water deficiency constrains P_N_ by acting both on CO_2_ supply and CO_2_ demand functions [90]. The first is driven by diffusive factors (i.e., stomatal and mesophyll conductance), and the latter is affected by factors operating at a metabolic level [91,92,93]. Under mild water stress, the photosynthetic CO_2_ uptake of plants is mainly limited by stomata closure [91,92,93,94]. A first signal of stomatal limitation is given by the increase in IWUE, which reveals an effective stomatal control on P_N_ caused by a sizable g_s_ decrease [95,96,97]. Our data showed that AA was not able to maximize IWUE, as this parameter did not significantly vary over the experiment, although it had a tendency to increase in response to the initial stress, due to a higher reduction of g_s_ than of P_N_. However, our results on the g_s_ decrease were substantially in accordance with Trifilò et al. [69], who reported the g_s_ decreasing at a similar extent in response to a moderate water stress.

Plant capacity to increase IWUE is viewed as a strategy to photosynthesize by optimizing evaporative water loss. Indeed, a lower g_s_ leads to a reduced transpiration rate, which in turn improves WUE, a key factor for plant growth under drought, as a higher WUE allows an effective carbon assimilation by reducing water consumption [98]. Petruzellis et al. [22] reported that, from a hydraulic point of view, AA is characterized by efficient water transport from roots to leaves. Thus, AA endures water stress of mild intensity without requiring an adjustment to limit E because the water lost by transpiration is rapidly replaced. On the other hand, the efficient water transport could also contribute to explain the maintenance of a fairly stable RWC_md_ besides morphological leaf traits. However, as water stress progressed, AA was not able to sustain further water loss without seriously compromising P_N_. Starting from the sixth day, a strong P_N_ impairment occurred simultaneously with maximum water stress, leading to lethal values. Indeed, according to Gulías et al. [89] and Gratani and Varone [86], when photosynthetic rates drop below 70% of the maximum, the P_N_ recovery to unstressed values may be compromised.

Over the experiment, RP showed a lower endurance to water stress, as its leaf water status appeared critical already from the first days of the water stress when ψ_md_ and RWC_md_ were two-fold lower than on the first day, reaching then on the sixth day remarkably low values.

The results of the effect size analysis corroborate a lower resistance to water stress of RP. In particular, RP showed the greatest variation of the effect size indices between unstressed conditions (i.e., on the first day) and the end of the experiment (i.e., on the ninth day) for all traits analyzed, except for LMA, a trait that strictly affects P_N_ and E [54]. Indeed, a lower water stress resistance in RP than in AA was consistent with LMA values, which, in RP, under an unstressed condition were 84% smaller than in AA. RP also showed less plasticity in adjusting LMA in response to water withholding, as resulted by the lack of significant differences between stressed and unstressed seedlings. After three days of water stress, RP achieved photosynthetic rates comparable to AA, but thereafter, on the sixth day, photosynthetic rates rapidly dropped to lethal values, i.e., 6% of the maximum P_N_. The initial capacity to maintain P_N_ rates equal to 70% of the maximum was due to a considerable g_s_ reduction, which in turn translated into a significant IWUE increase. However, the initial stomatal responsiveness seemed to be not enough for limiting considerably evaporative water losses, and then to maximize the CO_2_ assimilation. As confirmation, both E and WUE did not significantly vary between the first and the third day of water stress. The weaker stomatal control of E also contributed to the achievement of the very low ψ_md_ values observed on the third day of water stress. A more negative ψ_md_ was strictly related to an increase of the evaporative water losses, given that ψ_md_ was measured at midday when the temperature and irradiance and vapor pressure deficit reached the highest values [99]. Accordingly, ψ_md_ was considered a proxy for the daily maximum water stress [100]. Considering the RWC_md_ value at the third day of water stress, one may suppose that, besides stomatal limitation, P_N_ could be affected also by non-stomatal limitations. According to Lawlor and Cornic [101], RWC mirrors changes at a metabolic level, especially when it drops below 70%. At this level, impairments at a biochemical and photochemical level occur [45,91,93,102,103], and as a consequence, g_s_ was less effective in regulating P_N_ [101]. However, in this case, despite the very low RWC_md_, the hypothesis that non-stomatal limitations were at play is not very persuasive. Usually, metabolic limitations occur under severe water stress, and they are associated with both a strong IWUE and C_E_. In our case, on the third day of water stress, there was an increase of the IWUE and a slight decrease of C_E_, which was in line with the classical response driven by stomatal limitation [104,105].

The performance of PA under water stress in some respects was similar to AA and in others to RP.

In particular, although leaf water status was not particularly critical after three days of the stress imposition, such as AA, P_N_ similarly to RP rapidly decreased, reaching values corresponding to 54% of the maximum. The large P_N_ reduction on the third day occurred per se rather than as a consequence of stomatal closure, as P_N_ showed a higher decrease than g_s_. Moreover, PA showed a low plasticity in adjusting LMA in response to water withholding, showing likewise to RP no significant differences between unstressed and stressed seedlings.

Strategies based on water use maximizing are central to survive under Mediterranean climate conditions, especially in a climate change context. Overall, our results showed that the responses of AA, PA, and RP were not based on such a strategy because, essentially, these species displayed a lower stomatal control of P_N_, which is the first step to limit water lost. This could lead to supposing that AA, PA, and RP were able to endure water stress for a shorter time whether they are compared with species such as Mediterranean ones, which are evolutionary adapted to drought conditions. Indeed, from this point of view, the behavior of the considered IAS was comparable to some Mediterranean native species. For instance, Varone et al. [11], carrying out a similar induced-water stress experiment on Mediterranean evergreen species, found that saplings of *Rhamnus alaternus* and *Olea europaea* dropped their photosynthesis below 70% of the maximum after two and three days of water withholding, respectively. In addition, Varone and Gratani [42] reported that seedlings of *Cistus creticus*, *Rosmarinus officinalis*, and *Erica multiflora* reached the maximum water stress, with values of RWC and ψ similar to those obtained in this study, after nine days of the imposed water stress, with strong impairment already after six days.

On the other hand, it is commonly reported that drought-induced treatment increases IWUE. Our results compared with those obtained from Medrano et al. [105] for Mediterranean species highlighted a similar response of IAS during the no stress condition, whereas under the water stress condition, the IWUE of AA, PA, and RP was lower than the values reported for Mediterranean species [105], highlighting a water consumption behavior of AA, PA, and RP.

## 4. Materials and Methods

### 4.1. Study Site and Plant Material

The study was carried out at the Experimental Garden of Sapienza University of Rome (41°53′ N, 12°28′ E; 53 m a.s.l.) on seedlings of *Ailanthus altissima* (AA), *Phytolacca americana* (PA), and *Robinia pseudoacacia* (RP) obtained from seeds collected at the beginning of October 2018 from plants naturally growing in different public sites [106], comparable for conditions, in the city of Rome (Italy).

Seedlings were cultivated in black polyethylene plastic pots (14 cm diameter, 16 cm height, 2.5 L) filled with a basic cultivation substrate (COMPO Naturasol, Universal, Italy). The substrate was composed of neutral sphagnum peat, organic green soil improver, and pumice (pH = 7.0, electrical conductivity = 0.60 dS/m, dry bulk density = 210 Kg m^−3^, total porosity = 88%).

Seedlings were grown under a Mediterranean type of climate. According to data provided from Arsial Meteorological Station (Lanciani Street) for the period 2010–2020, the mean minimum air temperature of the coldest month (January) was 4.6 °C, the mean maximum air temperature of the hottest months (August) was 32.3 °C, and the annual mean air temperature was 17.0 °C. Total annual rainfall was 855.6 mm, most of which occurred in autumn and winter. The dry period was from June to August, characterized by a total rainfall of 108.9 mm.

### 4.2. Experimental Design

In June 2020, two-year-old seedlings were arranged in a randomized scheme on an area of 9 m^2^ covered by a white plastic sheet, which was laterally opened to allow air circulation and was arranged 2 m high to exclude rainfall during the experimental period. Seedlings were regularly watered to field capacity (measured by HydroSense II, Campbell Scientific, Logan, UT, USA) until the beginning of the induced water stress experiment on 1 July 2020, when gas exchange and leaf water status measurements were randomly carried out on seven seedlings per species. Thereafter, seedlings were randomly assigned to two treatments, i.e., water stress and control, each including 10 seedlings per species. Water stress treatment was induced by withholding water throughout the experiment. Leaf water parameter and gas exchange measurements on stressed plants were randomly carried out on 4–7 seedlings every 3 days. Data collected on the 1st sampling day, when all the plants were still well watered, corresponded to control values [11,42]. However, throughout the experiment, the control seedlings were kept under daily irrigation and measured to verify that the considered parameters maintained constant values [42], showing no significant differences for the duration of the experiment (from the first day to the ninth day).

The water stress experiment was stopped when the stomatal conductance in stressed plants was below 0.05 mol H_2_O m^−2^ s^−1^, which is indicative of a severe water stress condition [107]. At the end of the experiment, on 9 July, leaf morphological traits were measured on control and stressed seedlings.

During the experimental period from 1st to 9th of July, diurnal mean air temperature was 30.0 ± 3.1 °C, and air humidity was 51.6 ± 10.5%.

### 4.3. Leaf Water Status

Midday leaf water potential (ψ_md_, MPa) and relative water content (RWC_md_,%) were measured in each sampling occasion on two mature leaves per seedling per species. The ψ_md_ was measured by a pressure chamber (SKPM 1400 Skye Instruments, Llandrindod Wells, UK). The RWC_md_ was calculated as: RWC_md_ = (FM − DM)/(TM − DM) × 100, where FM was the leaf fresh mass, DM the leaf mass after oven drying at 90 °C until constant weight was reached, and TM the leaf mass after rehydration until saturation for 48 h at 5 °C in the darkness.

### 4.4. Gas Exchange Measurements

Gas exchange measurements included: net photosynthetic rate (P_N_ µmol CO_2_ m^−2^ s^−1^), stomatal conductance (g_s_, mol H_2_O m^−2^ s^−1^), transpiration rate (E, mmol H_2_O m^−2^ s^−1^), and sub-stomatal CO_2_ concentration (C_i_, ppm). Gas exchange and photosynthetically active radiation (PAR, µmol photon m^−2^ s^−1^) were measured by an open infrared CO_2_ gas analyzer (ADC–Lcpro, ADC, Hoddesdon, UK), equipped with a leaf chamber (PLC, ADC, Hoddesdon, UK). Measurements were carried out on cloud-free days (PAR > 1000 µmol photon m^−2^ s^−1^) in the morning (from 9.30 to 12.30) to ensure that near-maximum daily photosynthetic rates were measured [108]. On each sampling occasion, fully expanded leaves were used (two leaves per seedling per species). The instantaneous water use efficiency (WUE, µmol CO_2_ mol H_2_O^−1^), the intrinsic water use efficiency (IWUE, µmol CO_2_ mol^−1^ H_2_O), and the apparent carboxylation efficiency (C_E_, mol mol^−1^) were also calculated as: WUE = P_N_/E, IWUE = P_N_/gs and C_E_ = P_N_/C_i_.

### 4.5. Leaf Morphology

Morphological leaf traits, which included leaf area (LA, cm^2^) and leaf mass per area (LMA, mg cm^−2^) were measured on two fully expanded leaves per seedlings per species; seven control seedlings and seven stressed seedlings were randomly selected among the two treatments. The LA was measured on fresh leaves by an Image Analysis System (Delta–T Devices, Cambridge, UK); The LMA was calculated as a ratio between DM and LA.

### 4.6. Data Analysis

Two-way ANOVA was performed to test for main factors, namely species and sampling day, which were considered an indicators of water stress intensity, and their interaction (species × sampling day) on leaf water status and gas exchange parameters. Differences in leaf morphological traits between the control and stressed seedlings were also analyzed by two-ways ANOVA (main factors: species and treatment, interactive factor: species × treatment). Multiple comparisons were made by the post-hoc Tukey’s test. Moreover, Pearson’s linear correlation analysis was carried out to explore significant relationship among the considered physiological parameters. Statistical tests were performed using the statistical software PAST 4.07 [109] (https://www.nhm.uio.no/english/research/infrastructure/past/, accessed on 8 September 2021), and the significance level was fixed at *p* ≤ 0.05.

To assess the magnitude of the water stress responses, two effect size indices for standardized differences were computed: Hedges’ g and Glass’s delta.

The most commonly used effect size index is Cohen’s d, but Hedges’ g provides a bias correction (using the exact method) to Cohen’s d for small sample sizes. For sample sizes > 20, the results for both statistics are roughly equivalent. Hedges’ g is the estimated standardized difference between the means of two populations. Hedges’ g, in our case, was computed for each trait as the difference between trait means in each treatment divided by the pooled standard deviation [110]. However, Glass’s delta is appropriate when the standard deviations are significantly different between treatments, as it uses only the second group’s standard deviation.

Analysis was performed using the package effectsize [111] (R version 4.1.1).

Data collected in each sampling day per each species were shown as mean ± standard deviation (*n* = 4–7 seedlings).

## 5. Concluding Remarks

Our study pointed out that, despite a common strategy, AA, PA, and RP differ in their sensitivity to cope with water stress. In particular, even if AA was characterized by a more water-spending strategy, it was more resistant to water stress than PA and RP. In this view, the key factor was its higher LMA and especially its plasticity to increase LMA under stress conditions.

Drought tolerance of the considered species seemed to reflect the native climate and occupied habitats, confirming our hypothesis. In particular, AA comes from subtropical or warm temperate climates with long and warm growing seasons [69,112], while PA and RP grow in more mesic climates [76,81]. Accordingly, AA was the species that could better withstand an increase in drought conditions than PA and RP.

Further studies, such as experiments based on repeated water stress cycles, need to be carried out to evaluate the recovery capacity of these species and, therefore, to better clarify their response to water stress. In fact, following some observations carried out at the end of our experiment, a complete loss of RP leaves was observed, but, subsequently after a few weeks, the production of new leaves was observed (data no shown).

On the base of water stress tolerance, AA, PA, and RP could be good competitors of Mediterranean species, as forecasted aridity increase in this area could further increase their invasiveness. Indeed, in a study conducted on IAS and native species, Godoy et al. [113] showed that future scenarios of increased aridity in Mediterranean-type ecosystems associated with climate change will filter invasion success by taxonomic identity and will reveal the importance of studying ecophysiological traits to understand and better predict future biological invasions.

## Figures and Tables

**Table 1:** F values from two-way ANOVA carried out to test the effect of the water stress of species, sampling day, and their interaction (species × sampling day) on net photosynthetic rate (P_N_), water use efficiency (WUE), intrinsic water use efficiency (IWUE), relative water content (RWC_md_), leaf water potential (Ψ_md_), and apparent carboxylation efficiency (C_E_). ** *p* ≤ 0.001; * *p* ≤ 0.05.

Variable	Species	Sampling Day	Species × Sampling Day
PN	F = 244.0 **	F = 589.7 **	F = 36.7 **
WUE	F = 73.69 **	F = 33.4 **	F = 9.2 **
IWUE	F = 31.72 **	F = 3.7 *	F = 17.1 **
RWC_md_	F = 146.2 **	F = 393.3 **	F = 21.3 **
Ψ_md_	F = 461.2 **	F = 1602.0 **	F = 203.0 **
C_E_	F = 173.5 **	F = 351.9 **	F = 25.0 **

**Table 2:** Leaf morphology of *Ailanthus altissima* (AA), *Phytolacca americana* (PA), and *Robinia pseudoacacia* (RP) between the control seedling (c) and stressed seedling (s). Leaf area (LA) and leaf mass per area (LMA). Lowercase letters indicate differences within species, and capital letters indicate differences among species. Mean ± standard deviation with the same letter is not significantly different (ANOVA, *p* ≤ 0.005).

Species	LA (cm^2^)		LMA (mg cm^−2^)	
	c	s	c	s
AA	136.58 ± 26.25 aA	105.39 ± 19.23 bA	6.11 ± 0.98 aA	7.43 ± 1.12 bA
PA	65.58 ± 7.01 aB	47.98 ± 8.56 aB	3.32 ± 0.56 aB	3.91 ± 0.38 aB
RP	56.42 ± 20.97 aB	34.69 ± 8.11 aB	3.72 ± 0.60 aB	4.64 ± 0.89 aB
